# A facile preparation method for homogeneous Ir catalysts for dehydrogenation and hydrogenation reactions involving N-heteroarenes

**DOI:** 10.1039/d5ra05183e

**Published:** 2025-09-16

**Authors:** Xufeng Lin, Yixuan Zhang, Wenxu Feng, Qing Li, Yanyan Xi

**Affiliations:** a College of Chemistry and Chemical Engineering, China University of Petroleum (East China) Qingdao P. R. China 266580 hatrick2009@upc.edu.cn; b State Key Laboratory of Heavy Oil Processing, China University of Petroleum (East China) Qingdao P. R. China 266580; c Shandong Iron & Steel Group Co., Ltd Jinan P. R. China 250101; d Advanced Chemical Engineering and Energy Materials Research Center, China University of Petroleum (East China) Qingdao P. R. China 266580

## Abstract

The liquid organic hydrogen carrier (LOHC) technology is a promising route for efficient hydrogen storage, and Ir complexes belong to an important family of homogeneous catalysts applied in this field. We developed and optimized a “2-step” method for the synthesis of complex Cp*Ir(C_6_H_3_NF_3_O)Cl. This complex, designated as T1 in this work, was originally referred to as **2c** in ref. [Yamaguchi *et al. J. Am. Chem. Soc.*, 2009, **131**, 8410–8412]. The “2-step” method improved the yield of complex T1 and offered a simplified operational process, compared to the previously reported “sodium salt” method. The excellent catalytic activity of T1, synthesized by the “2-step” method was further confirmed. This method was further applied to synthesize other Ir catalysts, T2 and T3. 5-Fluoro-2-hydroxypyridine and 5-nitro-2-hydroxypyridine were assigned to prepare the dehydrogenation catalysts T2 and T3, respectively. Both T2 and T3 effectively catalyzed the dehydrogenation of 2-Me-THQ. Their catalytic activities followed the trend T1 > T2 > T3, highlighting the crucial role of electron-withdrawing ligands in enhancing catalytic performance. In addition, the precursor [Cp*IrHCl]_2_ (H1) obtained through this “2-step” method, exhibited good performance in 2-Me-Q hydrogenation. H1 and T1 were also successfully applied to the hydrogenation and dehydrogenation of other nitrogen-containing carriers, respectively.

## Abstract 2

A simple and efficient synthetic method was developed for preparing organometallic Ir complexes for catalysis in hydrogen storage processes.

## Graphical abstract

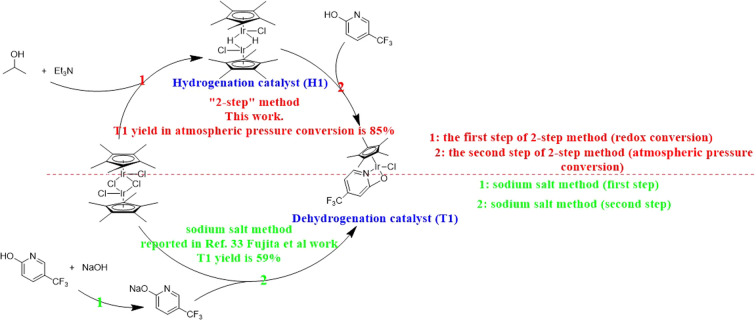

## 1. Introduction

Hydrogen energy is expected to play a significant role in future sustainable energy systems, as it can mitigate environmental pollution caused by fossil fuel consumption.^1–8^ However, the storage and transportation of hydrogen hinder its widespread application. Among various storage and transportation methods, such as metal hydrides,^9,10^ compressed gas hydrogen storage,^11,12^ and hydrogen liquefaction storage,^13,14^ liquid organic hydrogen carrier (LOHC) technology attracts researchers' interest due to its safety, promising hydrogen storage ability, low transportation expenses, and compatibility with existing infrastructures.^15,16^ This technology operates through the reversible hydrogenation–dehydrogenation cycle: hydrogenation of a hydrogen-lean organic carrier (H^−^-LOHC); and subsequently dehydrogenation of the resulting hydrogen-rich counterpart (H^+^-LOHC). Compared with traditional H^−^-LOHC/(H^+^-LOHC) carriers,^17–19^ nitrogen-containing carriers, such as 1,2-dimethylindole/octahydro-1,2-dimethylindole, *N*-ethylcarbazole/dodecahydro-*N*-ethylcarbazole and 2-(*N*-methylbenzyl)pyridine/perhydro-2-(*N*-methylbenzyl)pyridine have received increasing attention in recent years.^20–23^ This growing interest is due to their advantages such as a moderate dehydrogenation temperature, moderate reaction enthalpy, excellent hydrogen storage capacity, and stability.^24–26^

Undoubtedly, the performance of catalysts is crucial for the application of LOHC technology. Numerous studies have focused on heterogeneous catalysts for LOHC technology application. In contrast, research on homogeneous catalysts is rather sparse. However, studies have shown that homogeneous catalysts possess obvious advantages, such as a well-defined reaction pathway, excellent catalytic activity, and high product selectivity. Notably, Ir-based complexes exhibit good performance in the hydrogenation and dehydrogenation of nitrogen-containing compounds. Jensen *et al.*^27^ synthesized three different “PCP pincer Ir catalysts”, and the dehydrogenation results revealed that dehydrogenation to the fully unsaturated ethylcarbazole was difficult using the “PCP pincer Ir catalysts”. Riisager *et al.*^28^ investigated 2-methylindoline dehydrogenation using cationic Ir complexes with molten [PPh_4_][NTf_2_], achieving 100% dehydrogenation in 7 h at 140 °C. Fujita *et al.*^29^ developed a 2,5-dimethylpyrazine/2,5-dimethylpiperazine-based hydrogen storage system, repeating four times the hydrogenation–dehydrogenation cycle. Choudhury *et al.*^30^ reported that homogeneous Ir(iii)–NHC catalyst exhibited high stability and activity in dehydrogenation reaction. A DFT study showed that the dissociation and formation of H_2_ were the rate-determining step for hydrogenation and dehydrogenation, respectively. Qu *et al.*^31^ proposed an outer-sphere mechanism for Ir complex catalyzed pyridine hydrogenation reaction. Zhang and Xi^32^ studied the dehydrogenation reaction of THQ catalyzed by Cp*Ir complexes and then proposed a ligand cooperative pathway. They supposed that the dehydrogenation of THQ begins with the ligand-assisted cleavage of N–H bond by a Cp*Ir complex.

**Scheme 1:**
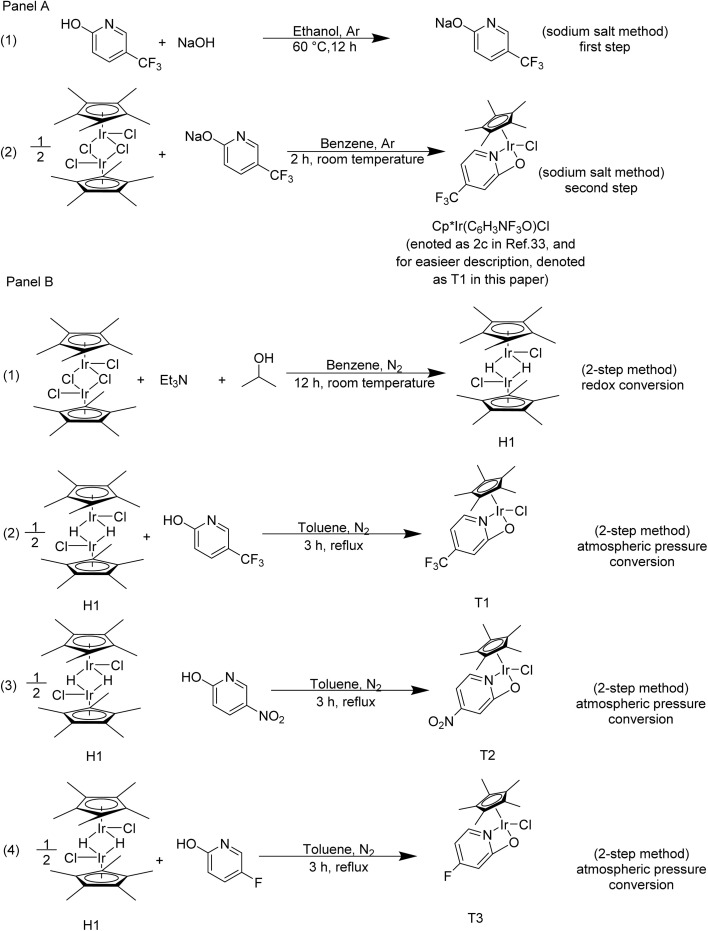
The synthesis of the Cp*Ir complexes. Panel A depicts the synthetic process of Cp*Ir(C_6_H_3_NF_3_O)Cl (complex **2c** in ref. 33, denoted as T1 in this paper) with the “sodium salt” method. Panel B depicts the synthesis process of T1, T2, T3 with a “2-step” method developed in this paper.

Cp*Ir(C_6_H_3_NF_3_O)Cl (denoted as **2c** in ref. 33, and for easier description, denoted as T1 in this paper) reported by Fujita *et al.*^33^ presented fairly good catalytic activity in THQ dehydrogenation. They employed a “sodium salt” method to synthesize a series of Ir complexes, such as T1 (see Panel A in Scheme 1). The synthesis process of the T1 by “sodium salt” method was divided into two steps. The first step was to prepare sodium salt under an Ar atmosphere, and the second step was to convert [Cp*IrCl_2_]_2_ to the T1 in an Ar atmosphere. The low solubility of the sodium salt in the organic phase was a major factor contributing to the moderate T1 yield of 59% (Panel A in Scheme 1). Subsequently, Fujita *et al.*^33^ mainly studied the dehydrogenation of THQ catalyzed by Cp*Ir complexes featuring an electron-donating ligand. And they barely investigated the dehydrogenation of THQ catalyzed by Cp*Ir complexes featuring an electron-withdrawing ligand.

Additionally, the poor solubility of sodium salt in the organic phase resulted in a moderate T1 yield. To address this issue, we developed an alternative “2-step” method. The “2-step” method was also effective for the synthesis of other Ir complexes. The synthesis of the Ir complexes *via* a “2-step” method was depicted in Panel B of Scheme 1. This method consisted of a redox conversion and an atmospheric pressure conversion. The yield of the T1 in atmospheric pressure conversion (Panel B (2) in Scheme 1) was 85% which was higher than that in the “sodium salt” method. Subsequently, the amount of solvent and type of alkaline additives were investigated in the T1 catalyzed 2-Me-THQ dehydrogenation reaction. Similarly, the catalytic activity of T2 and T3 was also investigated in the 2-Me-THQ dehydrogenation reaction. Fujita *et al.* reported the good catalytic activity of H1 in the 2-Me-Q hydrogenation. This complex was converted from T1 under a H_2_ atmosphere. Building on this, the influence of reaction temperature and H1 loading was further investigated. Catalysts H1 and T1 were also employed in other nitrogen-containing carrier hydrogenation and dehydrogenation reaction, respectively. Developing the synthetic strategy of Cp*Ir complex and improving its catalytic activity significantly deepens the understanding of homogeneous catalysts.

## 2. Results and discussion

### 2.1 The advantage of the “2-step” method

The detailed procedure of the “2-step” method, employed in the preparation of the Cp*Ir complexes, can be found in the Experimental section. A modification in our “2-step” method was the use of 2-hydroxy-5-trifluoromethylpyridine instead of the sodium 5-trifluoromethyl-2-pyridonate salt. This modification increased the yield of T1, because the solubility of 2-hydroxy-5-trifluoromethylpyridine was higher than that of sodium 5-trifluoromethyl-2-pyridonate salt in the organic phase. Furthermore, the “2-step” method was successfully extended to the preparation of T2 and T3 (*vide infra*), demonstrating its general applicability. In addition, complex [Cp*IrHCl]_2_ (H1) which couldn't be directly obtained *via* the “sodium salt” method,^33^ was successfully synthesized through the first step of the “2-step” method. The good catalytic activity of H1 was confirmed in the 2-Me-Q hydrogenation reaction.

### 2.2 Reaction parameter optimization for 2-Me-THQ dehydrogenation

#### 2.2.1 Effect of the amounts of the solvent

In a dehydrogenation process, the amounts of solvent play a role in modulating the reactant concentration. As shown in Table 1 (entries 1–5), the dehydrogenation performance of 2-Me-THQ catalyzed by complex T1 was evaluated using various amounts of solvent. The yield of 2-Me-Q exhibited a strong dependence on the amount of solvent. The dehydrogenation of 2-Me-THQ was carried out in the presence of complex T1 in *p*-xylene (2 mL), resulting in a 2-Me-Q yield of 49.35%. The yield of 2-Me-Q increased from 49.35 to 57.18% and then to a maximum of 94.73% as the volume of *p*-xylene was raised from 2 to 4 mL and then to 6 mL. With the *p*-xylene amount increased to 8 mL, the 2-Me-Q yield reduced to 59.53%. With 10 mL of *p*-xylene, the yield of 2-Me-Q was 21.42%. When 2 mL and 4 mL of *p*-xylene were added (in separate experiments), a large amount of black precipitate was observed upon the completion of the dehydrogenation reaction. Researchers have suggested several potential deactivation mechanisms, including ligand decomposition, metal deposition, the formation of cluster cations, and dimerization.^34–37^ However, the detailed deactivation mechanisms of the Cp*Ir complex are still unclear. Additionally, researchers have put forward the dehydrogenation mechanism of THQ catalyzed by the Cp*Ir complex.^32,33,38^ By integrating these previous findings on deactivation with the proposed dehydrogenation mechanism, we hypothesized that the formation of the black precipitate was associated with catalyst deactivation. The low solvent volume and high reaction temperature facilitate the cleavage of the Ir-ligand bond and then accelerate the deposition of Ir species, leading to the formation of black precipitate.

**Table 1:** Screening of dehydrogenation protocol^a^

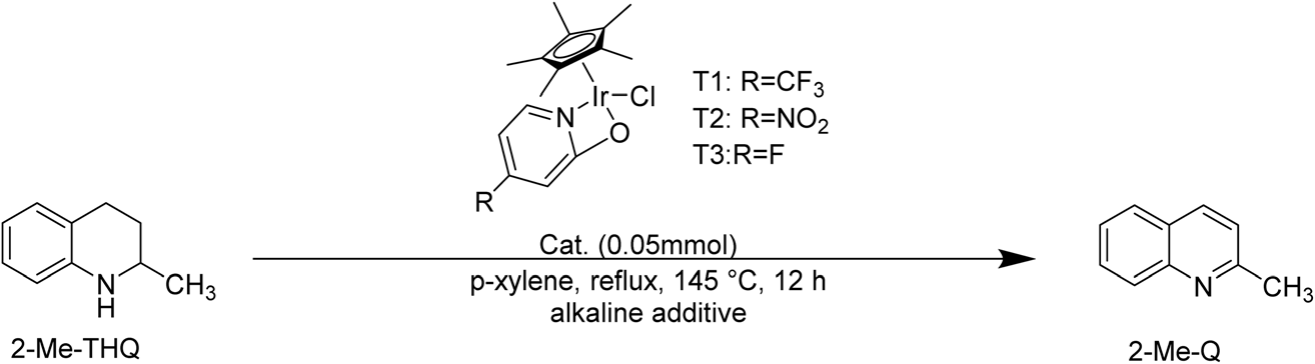				
Entry	Catalyst	Solvent (mL)	Alkaline additives	Yield^b^ (%)
1	T1	*p*-Xylene/2.0	—	49.35
2	T1	*p*-Xylene/4.0	—	57.18
3	T1	*p*-Xylene/6.0	—	94.73
4	T1	*p*-Xylene/8.0	—	59.53
5	T1	*p*-Xylene/10.0	—	21.42
6	T1	*p*-Xylene/6.0	Hexamethylphosphoramide	83.60
7	T1	*p*-Xylene/6.0	*N*-Methylpyrrolidone	83.10
8	T1	*p*-Xylene/6.0	1,4-Diazabicyclo[2.2.2]octane	30.72
9	T1	*p*-Xylene/6.0	Diethanolamine	22.52
10	T2	*p*-Xylene/6.0	—	90.32
11	T3	*p*-Xylene/6.0	—	88.21

a General reaction conditions: 2-Me-THQ (2.5 mmol), *p*-xylene (6 mL), TX (0.05 mmol), 12 h, 145 °C.

b The corresponding dehydrogenated products' yield was obtained by GC, and dodecane worked as an internal standard.

#### 2.2.2 Effect of the dosage of alkaline additives

Based on the optimal amount of *p*-xylene, we further investigated the dehydrogenation of 2-Me-THQ catalyzed by T1 in the presence of different alkaline additives (entries 6–9, Table 1). The 2-Me-Q yield of 94.73% was achieved in the absence of an alkaline additive. However, a decrease in 2-Me-Q yield was observed upon the addition of an alkaline additive. For instance, the addition of diethanolamine resulted in a dramatic decrease in 2-Me-Q yield. The 2-Me-Q yield was reduced to 30.72% with 1,4-diazabicyclo[2.2.2]octane added to the reaction system. The 2-Me-Q yield had a slight decrease to 83.60% and 83.10%, when hexamethylphosphoramide and *N*-methylpyrrolidone were used as additives, respectively.

Mitrichev *et al.*^39^ put forward a deactivation pathway of [Cp*IrCl_2_]_2_ in isopropyl alcohol in the presence of potassium *tert*-butoxide. The alkoxide replaced chloride ligands and then crowded anionic Ir(iii) complex with potassium counterions, resulting in the deactivation of [Cp*IrCl_2_]_2_. The role of amines in the 2-phenylquinoxaline hydrogenation was investigated by Mashima *et al.*^40^ detecting that the 2-phenylquinoxaline hydrogenation was difficult with the high-alkaline alkyl amines addition. The poor hydrogenation performance was perhaps related to the strong coordination between the alkyl amines and the Ir active site. Researchers also discovered that the poor dehydrogenation performance of THQ catalyzed by Fe complex might relate to the addition of KO^*t*^Bu.^41^ However, the introduction of alkaline additives in 2-Me-THQ dehydrogenation catalyzed by Ir complexes has rarely been explored. In the DFT studies, the dehydrogenation of 2-Me-THQ catalyzed by complex T1 proceeded *via* the ligand transformation, hydroxypyridine converted into pyridinone.^32,38,42^ The transformation from hydroxypyridine to pyridinone occurred through an intramolecular proton transfer.^32,38,42^ The introduction of alkaline additives hindered intramolecular proton transfer, impeding the formation of the Ir site, and inhibiting the dehydrogenation reaction. Similarly, beyond the intramolecular proton transfer pathway, the strong coordination between the Ir site and the additive reduced the number of catalytic Ir sites, resulting in the poor dehydrogenation performance of 2-Me-THQ.

### 2.3 Extending the “2-step” synthetic method to other Ir complexes

Electron-donating and electron-withdrawing ligands influence the electronic properties of complex, thereby affecting the dehydrogenation performance. Fujita *et al.*^33^ evaluated the catalytic activity of the Cp*Ir complexes, discovering that the catalytic activity decreased for complexes featuring electron-donating ligands. However, the behavior of complexes featuring electron-withdrawing ligands have been insufficiently explored. Therefore, using a “2-step” method, we successfully synthesized T2 and T3 with the 5-fluoro-2-hydroxypyridine and 5-nitro-2-hydroxypyridine, respectively. T2 and T3 both showed desirable catalytic activity for the 2-Me-THQ dehydrogenation (entries 10–11, Table 1). The yield of 2-Me-Q was 90.32% when 2-Me-THQ dehydrogenation catalyzed by T2 and the yield of 2-Me-Q was 88.21% when 2-Me-THQ dehydrogenation catalyzed by T3. The yield of 2-Me-Q decreased as the electron-withdrawing strength of the ligands diminished. Li *et al.*^38^ studied the catalytic mechanisms of the dehydrogenation of THQ catalyzed by T1. They subsequently found that introducing an electron-withdrawing –CF_3_ group into the complex lowered the ligand rotation barrier, benefiting the dehydrogeantion of THQ.^32,38^ Given the structural similarity between THQ and 2-Me-THQ, it was speculated that the dehydrogenation of 2-Me-THQ catalyzed by T1 proceeded through a similar mechanism. Consequently, we proposed that incorporating electron-withdrawing ligands into Cp*Ir complexes significantly enhances their catalytic efficacy in 2-Me-THQ dehydrogenation. An increase in the electron-withdrawing ability of the ligands led to a corresponding enhancement in 2-Me-THQ dehydrogenation performance.

### 2.4 Using T1 catalyst to other dehydrogenation substrates

**Scheme 2:**
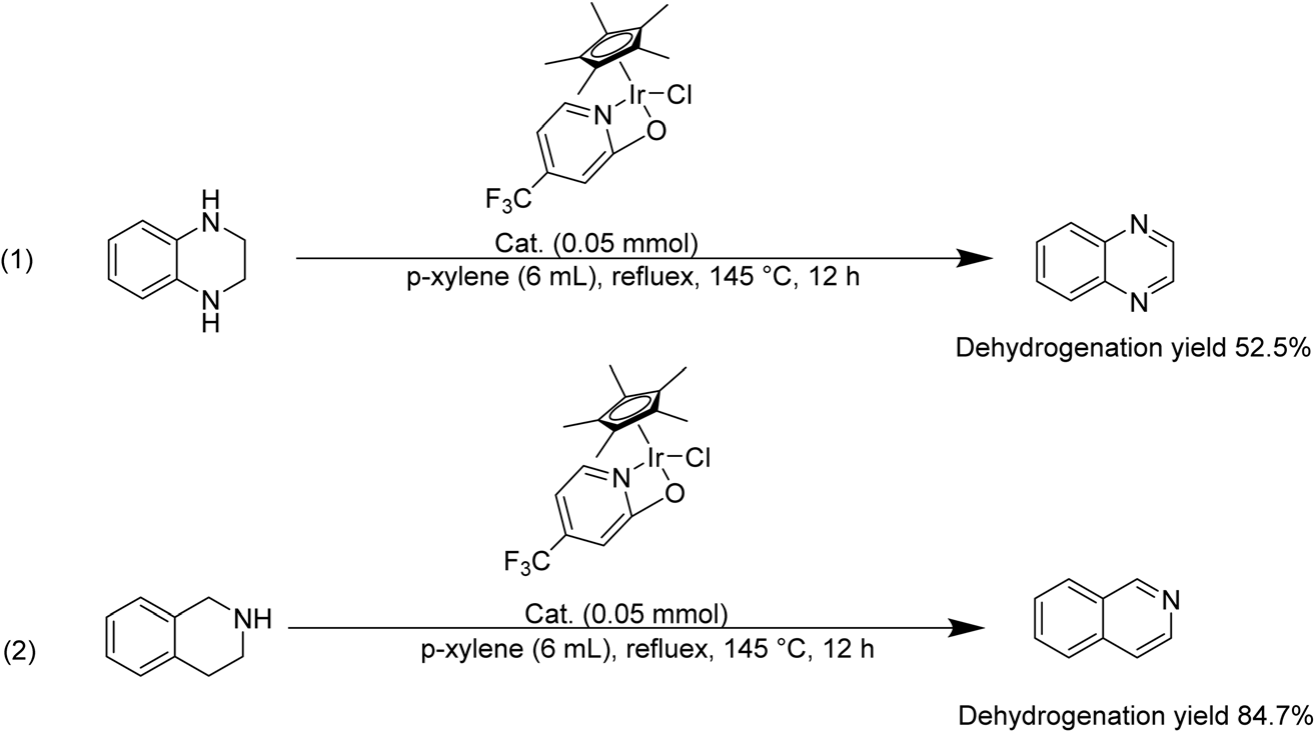
Examples of tetrahydro-*N*-heteroarene dehydrogenation catalyzed by T1.

Ideal LOHC substrates should possess the capacity for reversible hydrogenation and dehydrogenation while maintaining its frame work stability. Ref. 33 mainly compared the dehydrogenation performance of 2-Me-THQ, 3-Me-THQ, 4-Me-THQ, and 6-Me-THQ, discovering that 2-Me-THQ showed the optimum dehydrogenation performance. In order to broaden the range of nitrogen-containing candidates, tetrahydroquinoxaline and tetrahydroisoquinoline dehydrogenation reaction were carried out in the presence of T1 at 145 °C, 12 h (Scheme 2). The yield of isoquinoline was higher than that of quinoxaline. Oestreich and Yin^43^ pointed out that the dehydrogenation of N-heteroarene occurs gradually, involving the formation and the isomerization of imine intermediates. The dehydrogenation of tetrahydroquinoxaline followed the similar dehydrogenation pathway.^30^ The dehydrogenation of tetrahydroisoquinoline and tetrahydroquinoxaline catalyzed by Co complex, Fe complex, Ir complex have been reported.^41,44,45^ However, researchers mainly focus on evaluating the catalytic activity, and barely discuss the reason for the different dehydrogenation performance between tetrahydroisoquinoline and tetrahydroquinoxaline. According to the above-mentioned reports, the different dehydrogenation performance of tetrahydroisoquinoline and tetrahydroquinoxaline might relate to their alkalinity. Compared hydroisoquinoline with hydroquinoline, the former was more alkaline and thus more likely to result in Ir active site poisoning.^46–49^ Compared tetrahydroisoquinoline with tetrahydroquinoxaline, the latter owned two sp^3^ hybridization N atoms along with two lone-pair electrons, the origin of alkaline. The alkaline of tetrahydroquinoxaline from lone-pair electrons higher than that in tetrahydroisoquinoline.

### 2.5 Reaction parameter optimization for 2-Me-Q hydrogenation

#### 2.5.1 Effect of the temperature of the hydrogenation reaction

In the previous sections, the dehydrogenation of 2-Me-THQ as a H^+^-LOHC was systematically investigated. As mentioned in the ref. 33, the H1 complex, a hydrogenation catalyst, can be *in situ* generated from T1 at the presence of H_2_ atmosphere.^33^ In this work, the complex H1 was synthesized in the first step of the “2-step” method and then separated. Subsequently, the catalytic performance of separated H1 deserve to be further investigate.

The hydrogenation of 2-Me-Q, catalyzed by H1 was evaluated at four different temperatures under a 0.8 MPa H_2_ pressure in *p*-xylene. The yield of 2-Me-THQ presented a step-by-step enhancement from 10.99% at 60 °C to 99.26% at 120 °C with the temperature range from 60 °C to 120 °C (entries 1–4, Table 2). The yield of 2-Me-THQ was 92.50% at 80 °C, and the yield was 94.81% at 100 °C. Undoubtedly, increasing the reaction temperature raised the collision frequency between 2-Me-Q and H1, thereby facilitating 2-Me-Q hydrogenation. However, some black precipitates appeared under 120 °C in 8 h at a reducing ambient. Researchers supposed that [Cp*IrCl_2_]_2_ deactivations can occur by over-reaction with alkoxide to replace both chloride ligands, and possibly by further reaction to give the sterically crowded anionic Ir(iii) complex with potassium counte.^39^ The deactivation pathway of homogeneous catalysts includes ligand dissociation, dimerization, and metal deposition.^34–37^ According to the previous reports, we proposed that the formation of the black precipitates was associated with the dissociation of Cp ligand in complex H1.

**Table 2:** Screening of hydrogenation protocol^a^

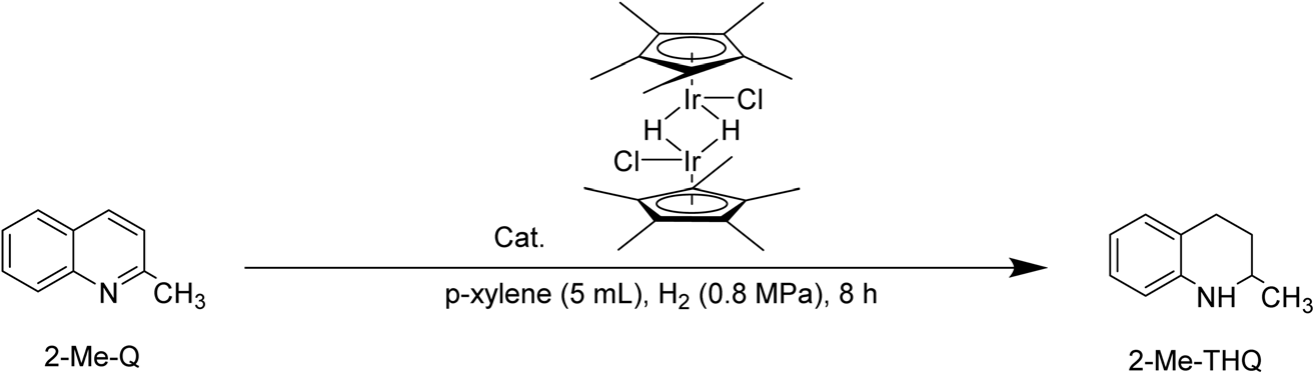				
Entry	Catalyst (mol%)	Solvent	Temperature (°C)	Yield^b^ (%)
1	H1/2.0	*p*-Xylene	60	10.99
2	H1/2.0	*p*-Xylene	80	92.50
3	H1/2.0	*p*-Xylene	100	94.81
4	H1/2.0	*p*-Xylene	120	99.26
5	H1/0.1	*p*-Xylene	120	1.09
6	H1/0.2	*p*-Xylene	120	4.30
7	H1/0.5	*p*-Xylene	120	61.05
8	H1/1.0	*p*-Xylene	120	89.43

a General reaction conditions: 2-Me-Q (2.5 mmol), *p*-xylene (5 mL), 8 h, H1 (2 mol%).

b The corresponding hydrogenated products' yield was obtained by GC, and dodecane worked as an internal standard.

#### 2.5.2 Effect of the dosage of the H1 catalyst

Researchers discovered that H1, converted from T1 during hydrogenation, played a real catalytic role in this reaction.^33^ Therefore, it was essential to research the 2-Me-Q hydrogenation performance with various loadings of H1. When the H1 dosage range from 2 mol% to 0.1 mol%, the yield of 2-Me-THQ presented a step-by-step decline from 99.26% with 2 mol% H1 complex to 1.09% with 0.1 mol% H1. (entries 4–8, Table 2). The yield of 2-Me-THQ was 89.43%, 61.05% and 4.30% under 1.0 mol%, 0.5 mol% and 0.2 mol% H1 complex addition, respectively. As the dosage of H1 increased, so did the activation of hydrogen and the concentration of the Ir–H species, facilitating the hydrogenation of 2-Me-Q.

### 2.6 Using H1 catalyst to other hydrogenation substrates

**Scheme 3:**
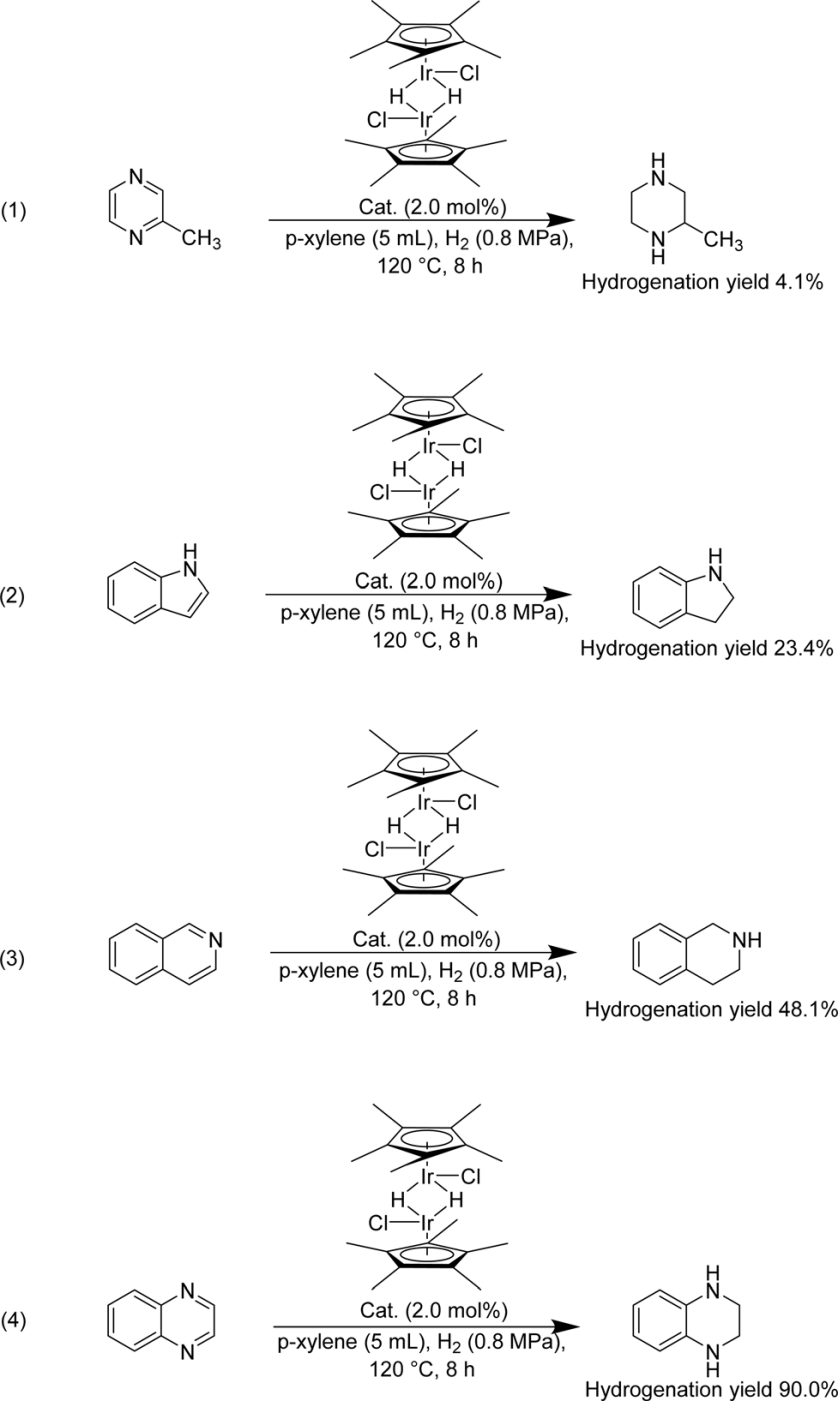
Hydrogenation of selected N-heteroarenes catalyzed by H1.

Certainly, the hydrogenation of other nitrogen-containing H^−^-LOHC was also essential to be investigated using H1. The hydrogenation performance of nitrogen-containing carriers, such as 2-methylpyrazine, indole, isoquinoline and quinoxaline was shown in Scheme 3. The hydrogenation of nitrogen-containing carriers was conducted at 120 °C for 8 h with 2 mol% H1 along with an H_2_ atmosphere. The hydrogenation yield of 2-methylpyrazine, indole and isoquinoline was 4.1%, 23.4% and 48.1%, respectively. And the hydrogenation yield of quinoxaline was 90%, the optimum hydrogenation yield. According to Beller *et al.*^47^ report, the quinoxaline was hydrogenated at 60 °C, while a higher temperature was required for isoquinoline hydrogenation. Dobereiner *et al.*^48^ investigated the hydrogenation pathway of 2-Me-THQ through DFT study. They put forward a stepwise outer-sphere mechanism, involving sequential proton and hydride transfer. In addition, they deemed that the substrate selectivity may be partially limited by p*K*_a_ differences between proton donor (the dihydrogen complex) and proton acceptor (substrates). Wang *et al.*^50^ investigated Pt nanoparticles catalyzed quinoline and isoquinoline hydrogenation, discovering that quinoline hydrogenation was more likely to occur than isoquinoline. Researchers mainly focus on expanding the range of suitable H^−^-LOHC, while discussion of their corresponding hydrogenation mechanisms is rarely. Therefore, we assume that the different hydrogenation performance might relate to their aromatic properties and alkalinity.^46,49^ The lone-pair electrons of N atoms in 2-methylpyrazine which are responsible for its basicity, didn't participate in the π-conjugated framework, reducing its hydrogenation performance. Indole, isoquinoline, quinoxaline, contained a benzene structure, an electron-donating group. The N atom in indole is sp^2^ hybridization and the lone-pair electron of the N atom participates in constitute the π-conjugated framework, possessing the weakest alkaline in these carriers. The results reported in the literature^46–49^ showed that the hydrogenation of isoquinoline was tough because of its special structure, for instant, isoquinoline possessed one sp^2^ N atom and the lone-pair electron of the N atom didn't participate in the π-conjugated framework. Compared to isoquinoline, quinoxaline possessed two sp^2^ N atom and the lone-pair electron of the N atom didn't participate in the π-conjugated framework, boosting the hydrogenation reaction.

## 3. Conclusion

The dehydrogenation catalyst Cp*Ir(C_6_H_3_NF_3_O)Cl (T1), was synthesized through a simple and efficient “2-step” method. The precursor complex [Cp*IrHCl]_2_ (H1), which can serve as a hydrogenation catalyst, was obtained in the first step of “2-step” method. Subsequently, H1 was converted to T1 under a N_2_ atmosphere by adding the ligand. Using “2-step” method, the yield of T1 was improved compared to the previous report. The high dehydrogenation activity of T1 in the dehydrogenation of 2-Me-THQ confirmed the effectiveness of the “2-step” method for synthesizing Cp*Ir complex. Following the same synthetic route, 5-fluoro-2-hydroxypyridine and 5-nitro-2-hydroxypyridine were used to synthesize other dehydrogenation catalysts, T2 and T3, respectively. The influence of the electron-withdrawing ligands was evaluated by comparing the dehydrogenation performance of 2-Me-THQ catalyzed by T1, T2, and T3. In addition, the effectiveness of the vital hydrogenation catalyst H1, was confirmed by its performance in the hydrogenation of 2-Me-Q. Finally, H1 and T1 were applied to the hydrogenation and dehydrogenation reactions, respectively, using other nitrogen-containing carriers. H1 was active for the quinoxaline hydrogenation and T1 was active for the tetrahydroisoquinoline dehydrogenation.

## 4. Experimental section

### 4.1 General

An Agilent 7820A GC system was applied to conduct gas chromatography analyses and dodecane served as an internal standard. ^1^H NMR tests were recorded in a Bruker AVANCE 500 spectrometer (CDCl_3_: *δ* = 7.26 ppm for ^1^H NMR spectra). Vario EL CUBE from Elementar were employed in analyzing element content.

### 4.2 Preparation of the catalyst

The complex T*X* (*X* = 1, 2, 3) and complex H1 were synthesized by a “2-step” method. The “2-step” method comprised redox conversion and atmospheric pressure conversion, as shown in Panel B of Scheme 1.

#### 4.2.1 Preparation of the H1

The redox conversion, Panel B (1) of Scheme 1, showed the synthesis of the precursor H1, which could catalyze H^−^-LOHC hydrogenation. The [CpIrCl_2_]_2_ (0.05 mmol) was placed in a 10 mL Schlenk flask. The reaction vessel was evacuated and refilled with N_2_ several times using a Schlenk line. Then, isopropanol (0.4 mL), triethylamine (0.1 mL), and benzene (1.0 mL) were added to the reaction vessel. The reactants were magnetically stirred at 25 °C for 12 h. The product was concentrated by rotary evaporation. After rotary evaporation, the residue was dissolved in benzene and then filtered. The filtrate was further concentrated by rotary evaporation. The sample was recrystallized several times from a benzene/cyclohexane mixture. Following washing with methanol, the sample was dried in *vacuo*. The ^1^H NMR and elemental analysis results of H1 are shown in SI.

#### 4.2.2 Preparation of the TX (*X* = 1, 2, 3)

The conversion of H1 to T*X* (*X* = 1, 2, 3), a step referred to as the atmospheric pressure conversion, was shown in Panel B of Scheme 1. The H1 (0.05 mmol) was taken in a 10 mL a Schlenk flask. The reaction vessel was evacuated and refilled with N_2_ several times using a Schlenk line. Then, 2-hydroxy-5-trifluoromethylpyridine (0.10 mmol) and toluene (3.0 mL) were injected into the Schlenk flask. The reactants were magnetically stirred and heated for 3 h. The product was concentrated by rotary evaporation. After rotary evaporation, the residue was dissolved in benzene and then filtered. The filtrate was further concentrated by rotary evaporation. The sample was recrystallized several times from a benzene/cyclohexane mixture. Following washing with methanol, the sample was dried in *vacuo*. Following the same synthetic procedure, T2 and T3 were synthesized by substituting 2-hydroxy-5-trifluoromethylpyridine with 5-nitro-2-hydroxypyridine and 5-fluoro-2-hydroxypyridine, respectively (Panel B (3) of Scheme 1 and Panel B (4) of Scheme 1). The ^1^H NMR and elemental analysis results of T1, T2 and T3 are shown in SI.

### 4.3 The dehydrogenation of hydrogenated N-heteroarenes

The dehydrogenation reaction was performed in a round-bottom flask equipped with a reflux condenser. The reactant and catalyst were added to the flask with a ratio of 50 : 1. In addition, the *p*-xylene was introduced into the dehydrogenation system as a solvent. The dehydrogenation reaction was carried out at 145 °C for 12 h under magnetic stirring. The corresponding dehydrogenation yield was obtained by GC analysis. All the substrates in the dehydrogenation reaction were purchased.

### 4.4 The hydrogenation of N-heteroarenes

The hydrogenation reaction was performed in an autoclave reactor under 0.8 MPa H_2_. The residual air and humidity in the reactor were removed by purging H_2_ three times. 2.5 mmol reactant, *p*-xylene (5 mL) and H1 catalyst were added to the autoclave reactor. The corresponding hydrogenated yield was obtained by GC analysis. All the substrates in the hydrogenation reaction were purchased.

## Author contributions

Xufeng Lin: methodology, formal analysis, writing – review and editing, project administration, funding acquisition; Yixuan Zhang: data curation, formal analysis, investigation, methodology, writing – original draft; Wenxu Feng: data curation, formal analysis, investigation, methodology, writing – original draft; Qing Li: formal analysis, writing – review and editing; Yanyan Xi: formal analysis, supervision.

## Conflicts of interest

The authors declare no competing financial interest.

## Supplementary Material

RA-015-D5RA05183E-s001

## Data Availability

The data supporting this article have been included as part of the SI. Supplementary information: the synthesis of H1, T1, T2, T3. The dehydrogenation process of hydrogenated N-heteroarenes. The hydrogenation process of N-heteroarenes. The analysis method of hydrogenation and dehydrogenation reactions. Characterization of H1, T1, T2 and T3. See DOI: https://doi.org/10.1039/d5ra05183e.
