# Supplementary material for: A facile preparation method for homogeneous Ir catalysts for dehydrogenation and hydrogenation reactions involving N-heteroarenes
Source: RSC Adv. 2025 Sep 16;15(40):33750–7. doi: 10.1039/d5ra05183e (PMC12439334; doi:10.1039/d5ra05183e)
Supplement: RA-015-D5RA05183E-s001 [file RA-015-D5RA05183E-s001.pdf]

Supplementary Information (SI) for RSC Advances.
This journal is © The Royal Society of Chemistry 2025

Electronic Supplementary Information: A facile preparation method for homogeneous Ir catalysts for dehydrogenation and hydrogenation reactions involving N-heteroarenes

Xufeng Lin[1,2*], Yixuan Zhang[1], Wenxu Feng[1,3], Qing Li[1], Yanyan Xi[4]

[1]College of Chemistry and Chemical Engineering, China University of Petroleum (East China), Qingdao, P. R. China, 266580

[2]State Key Laboratory of Heavy Oil Processing, China University of Petroleum (East China), Qingdao, P. R. China, 266580

[3]Shandong Iron & Steel Group Co., Ltd, Jinan, P. R. China, 250101

[4]Advanced Chemical Engineering and Energy Materials Research Center, China University of Petroleum (East China), Qingdao, P. R. China, 266580

*Authors to whom correspondence should be addressed.

Email: hatrick2009@upc.edu.cn

Content

The synthesis of hydrogenation and dehydrogenation catalysts 3

Dehydrogenation of hydrogenated N-heteroarenes...5

Hydrogenation of N-heteroarenes 6

The analysis method of hydrogenation and dehydrogenation reactions 7

Characterization of H1, T1, T2, and T3 8

The ^{1}H NMR spectra and elemental analysis results of H1, T1, T2, and T3......................9

The synthesis of hydrogenation and dehydrogenation catalysts

The synthesis of hydrogenation catalyst H1

The redox conversion, Panel B (1) of Sch. 1, showed the synthesis of the precursor H1, which could catalyze H^--LOHC hydrogenation. The $[CpIrCl_2]_2$ (0.05 mmol) was placed in a 10 mL Schlenk flask. The reaction vessel was evacuated and refilled with N_2 several times using a Schlenk line. Then, isopropanol (0.4 mL), triethylamine (0.1 mL), and benzene (1.0 mL) were added to the reaction vessel. The reactants were magnetically stirred at 25 °C for 12 h. The product was concentrated by rotary evaporation. After rotary evaporation, the residue was dissolved in benzene and then filtered. The filtrate was further concentrated by rotary evaporation. The sample was recrystallized several times from a benzene/cyclohexane mixture. Following washing with methanol, the sample was dried in *vacuo*. The ^1H NMR and elemental analysis results of H1 are shown in supporting information.

H1:^1H NMR (500 MHz, $CDCl_3$): δ = 1.69(s,15H), 7.36(s,1H); element content: C 34.29%; H 4.78%.

The synthesis of dehydrogenation catalyst T1

The conversion of H1 to TX (X=1, 2, 3), a step referred to as the atmospheric pressure conversion, was shown in Panel B of Sch. 1. The H1 (0.05 mmol) was taken in a 10 mL a Schlenk flask. The reaction vessel was evacuated and refilled with N_2 several times using a schlenk line. Then, 2-hydroxy-5-trifluoromethylpyridine (0.10 mmol) and toluene (3.0 mL) were injected into the Schlenk flask. The reactants were magnetically stirred and heated for 3 h. The product was concentrated by rotary evaporation. After rotary evaporation, the residue was dissolved in benzene and then filtered. The filtrate was further concentrated by rotary evaporation. The sample was

recrystallized several times from a benzene/cyclohexane mixture. Following washing with methanol, the sample was dried in *vacuo*.

T1 yield was 85% in atmospheric pressure conversion.

T1: ^{1}H NMR (500 MHz, $CDCl_3$): δ=1.77(s,15H), 6.27(s,1H), 7.57(s,1H), 8.09(s,1H); element content: C 35.24%; H 3.13%; N 2.19%.

The synthesis of dehydrogenation catalyst T2

The conversion of H1 to TX (X=1, 2, 3), a step referred to as the atmospheric pressure conversion, was shown in Panel B of Sch. 1. The H1 (0.05 mmol) was taken in a 10 mL a Schlenk flask. The reaction vessel was evacuated and refilled with N_2 several times using a schlenk line. Then, 5-nitro-2-hydroxypyridine (0.10 mmol) and toluene (3.0 mL) were injected into the Schlenk flask. The reactants were magnetically stirred and heated for 3 h. The product was concentrated by rotary evaporation. After rotary evaporation, the residue was dissolved in benzene and then filtered. The filtrate was further concentrated by rotary evaporation. The sample was recrystallized several times from a benzene/cyclohexane mixture. Following washing with methanol, the sample was dried in *vacuo*.

T2 yield was 78% in atmospheric pressure conversion.

T2: ^{1}H NMR (500 MHz, $CDCl_3$): δ=1.74(s,15H), 6.14(s,1H), 8.20(s,1H), 8.89(s,1H); element content: C 34.27%, H 3.15%, N 5.11%.

The synthesis of dehydrogenation catalyst T3

The conversion of H1 to TX (X=1, 2, 3), a step referred to as the atmospheric pressure conversion, was shown in Panel B of Sch. 1. The H1 (0.05 mmol) was taken in a 10 mL a Schlenk flask. The reaction vessel was evacuated and refilled with N_2

several times using a schlenk line. Then, 5-fluoro-2-hydroxypyridine (0.10 mmol) and toluene (3.0 mL) were injected into the Schlenk flask. The reactants were magnetically stirred and heated for 3 h. The product was concentrated by rotary evaporation. After rotary evaporation, the residue was dissolved in benzene and then filtered. The filtrate was further concentrated by rotary evaporation. The sample was recrystallized several times from a benzene/cyclohexane mixture. Following washing with methanol, the sample was dried in *vacuo*.

T3 yield was 80% in atmospheric pressure conversion.

T3: ^{1}H NMR (500 MHz, $CDCl_3$): δ=1.73(s,15H), 6.16(s,1H), 7.17(s,1H), 7.71(s,1H); element content: C 36.41%; H 3.12%, N 2.67%.

Dehydrogenation of hydrogenated N-heteroarenes

The dehydrogenation reactant 2-methyl-1,2,3,4-tetrahydroquinoline, tetrahydroquinoxaline and tetrahydroisoquinoline was obtained from Shanghai Macklin Biochemical Co., Ltd, Shanghai Haohong Scientific Co., Ltd, and Shanghai Adamas Reagent Co., Ltd, respectively.

The dehydrogenation reaction was performed in a round-bottom flask equipped with a reflux condenser. The reactant and catalyst were added to the flask with a ratio of 50:1. In addition, the p-xylene was introduced into the dehydrogenation system as a solvent. The dehydrogenation reaction was carried out at 145 °C for 12 h under magnetic stirring. The corresponding dehydrogenation yield was obtained by GC analysis.

In the section 2.2.1, the dosage of the p-xylene was investigated, and the dosage of the p-xylene was 2.0 mL, 4.0 mL, 6.0 mL, and 8.0 mL, respectively.

In the section 2.2.2, the influence of the alkaline additives like, hexamethylphosphoramide, N-methylpyrrolidone, 1,4-Diazabicyclo [2.2.2] octane, diethanolamine, was investigated, maintaining a fixed p-xylene addition (6 mL).

The corresponding dehydrogenation yield was obtained by GC analysis through internal standard method. And the dodecane worked as an internal standard substance.

Hydrogenation of N-heteroarenes

2-methylquinoline (2-Me-Q) was obtained from Shanghai Macklin Biochemical Co., Ltd. 2-methylpyrazine was gained from Shanghai Aladdin Biochemical Technology Co., Ltd. Indole, isoquinoline and quinoxaline was all obtained from Shanghai Adamas Reagent Co., Ltd.

The hydrogenation reaction was performed in an autoclave reactor under 0.8 MPa H_2. The residual air and humidity in the reactor were removed by purging H_2 three times. 2.5 mmol reactant, p-xylene (5 mL) and H1 catalyst were added to the autoclave reactor. The corresponding hydrogenated yield was obtained by GC analysis.

In the section 2.5.1, the temperature of hydrogenation reaction was discussed, and the temperature was 60 °C, 80 °C, 100 °C, and 120 °C, respectively.

In the section 2.5.2, the dosage of the H1 catalyst was investigated, and the the dosage of the H1 was 0.1 mol%, 0.2 mol%, 0.5 mol%, 1.0 mol% and 2.0 mol%, maintaining hydrogenation temperature (120 °C).

The corresponding hydrogenated yield was obtained by GC analysis. And the dodecane worked as an internal standard substance. All the substrates in hydrogenation reaction were purchased.

The analysis method of hydrogenation and dehydrogenation reactions

The relative correction factor(f_i') was obtained from the following formula:

$$f_i' = \frac{m_i}{m_s} \times \frac{A_s}{A_i}$$

m_i is the mass of the target substance; m_s is the mass of the internal standard substance; A_i is the corresponding GC integrated area of the target substance; A_s is the corresponding GC integrated area of the internal standard substance.

The mass of the product was calculated through internal standard method with GC, and the dodecane worked as an internal standard substance. The calculation formula for product quality was as follows:

$$m_i = \frac{A_i f_i}{A_s f_s} \times m_s = \frac{A_i}{A_s} \times f_i' \times m_s$$

The formula of hydrogenation/dehydrogenation yield (γ) was as follows:

$$\gamma = \frac{m_p}{M_p} \times \frac{M_R}{m_R} \times \eta \times 100\%$$

m_p is the mass of the product; M_p is the relative molecular mass of the product; m_R is the mass of the reactant; M_R is the relative molecular mass of the reactant; η is the stoichiometric ratio of reactant to product.

Characterization of H1, T1, T2, and T3

The ^{1}H NMR characterization of the hydrogenation and dehydrogenation catalysts

The ^{1}H NMR characterization was conducted on a Bruker Avance 500 spectrometer, using chloroform-d ($CDCl_3$) as the solvent (δ = 7.26 ppm).

The elemental analysis of the hydrogenation and dehydrogenation catalysts

Elemental analysis was performed using a Elementar Vario EL CUBE instrument. The C, H, N, and S elements in samples were converted into detectable gases. Subsequently, the obtained gas mixture was separated and then quantified by the thermal conductivity detection (TCD).

The ^{1}H NMR spectra and elemental analysis results of H1, T1, T2, and T3

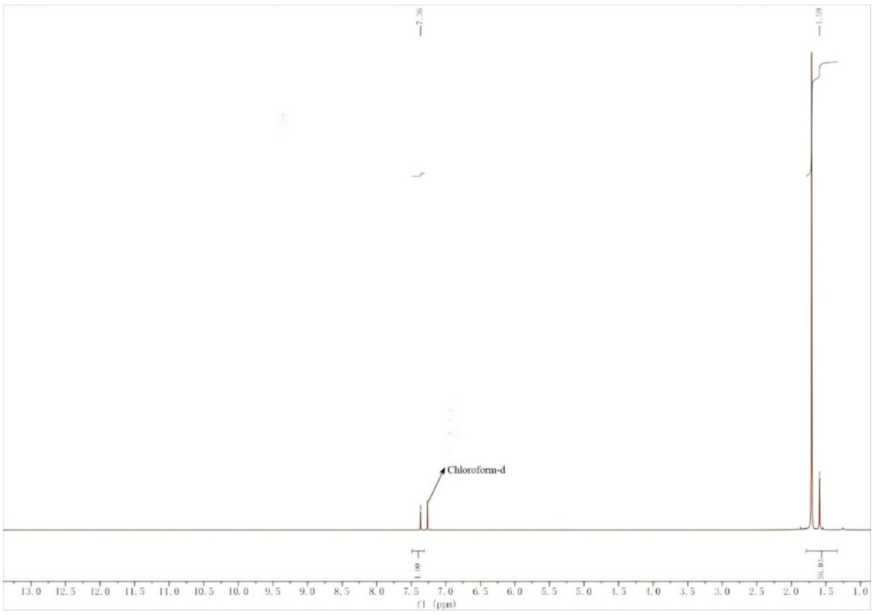

Figure S1 The ^{1}H NMR of H1

^{1}H NMR (500 MHz, $CDCl_3$): δ = 1.69(s,15H), 7.36(s,1H);
element content: C 34.29%; H 4.78%.

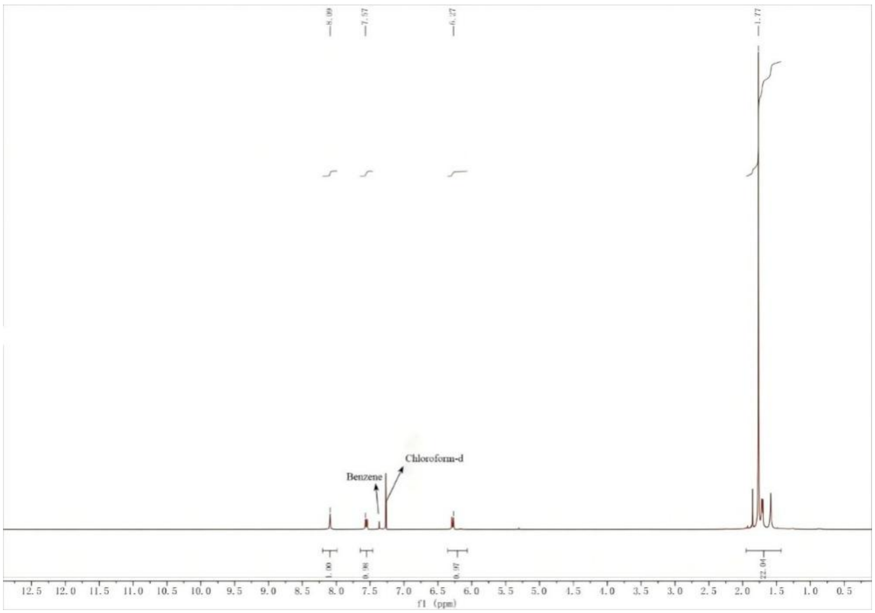

Figure S2 The ^{1}H NMR of T1

^{1}H NMR (500 MHz, $CDCl_3$): δ = 1.77(s,15H), 6.27(s,1H), 7.57(s,1H), 8.09(s,1H);
element content: C 35.24%; H 3.13%; N 2.19%

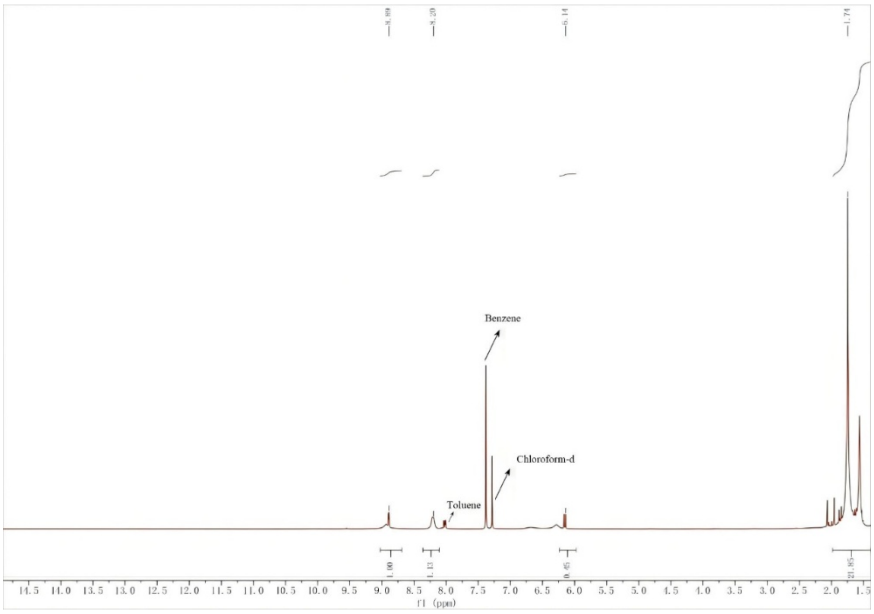

Figure S3 The ^{1}H NMR of T2

^{1}H NMR (500 MHz, $CDCl_3$): δ = 1.74(s,15H), 6.14(s,1H), 8.20(s,1H), 8.89(s,1H);

element content: C 34.27%, H 3.15%, N 5.11%

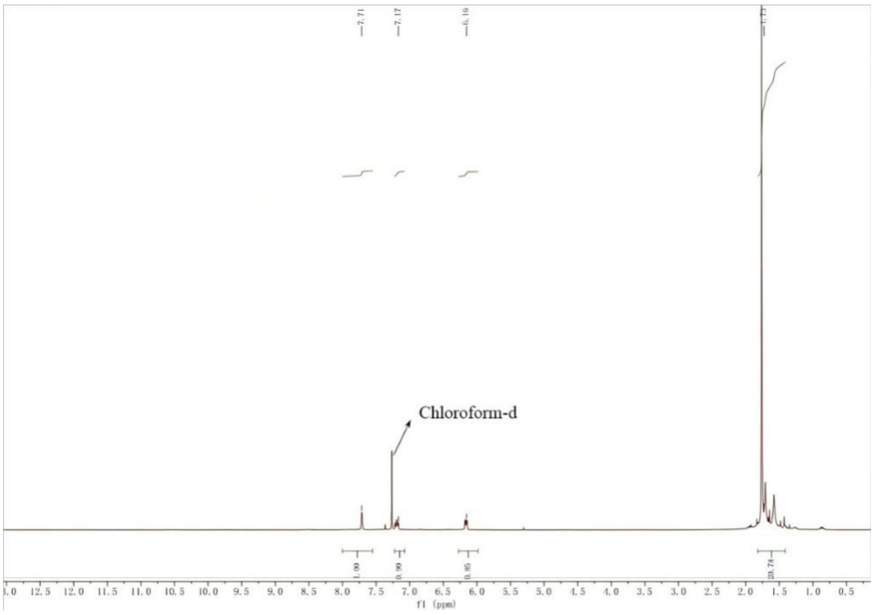

Figure S4 The ^{1}H NMR of T3

T3 ^{1}H NMR (500 MHz, $CDCl_3$): δ = 1.73(s,15H), 6.16(s,1H), 7.17(s,1H), 7.71(s,1H);
element content: C 36.41%; H 3.12%, N 2.67%
